# From Infarction to Angiosarcoma: Unraveling the Mystery of Pericardial Hematoma

**DOI:** 10.1016/j.jaccas.2025.103874

**Published:** 2025-05-14

**Authors:** Chunna Jin, Yuhua Wang, Zhanglong Hu, Dandan Huang, Su Lin, Xiaohong Gu, Jun Jiang

**Affiliations:** a Department of Cardiology, The Second Affiliated Hospital, Zhejiang University School of Medicine, Hangzhou, Zhejiang, China; b Department of Cardiovascular Surgery, The Second Affiliated Hospital, Zhejiang University School of Medicine, Hangzhou, Zhejiang, China; c Department of Cardiology, Medical Treatment Center of Ningbo Lihuili Hospital, Ningbo, Zhejiang, China; d Department of Cardiology, The First Affiliated Hospital of Ningbo University, Ningbo, Zhejiang, China

**Keywords:** cancer, imaging, myocardial infarction, pericardial effusion

## Abstract

**Background:**

Primary cardiac angiosarcomas are extremely rare, highly aggressive tumors characterized by rapid progression and high metastatic capability.

**Case Summary:**

We present a case with unexplained pericardial hematoma after repeated right coronary infarction, combined with multimodality imaging and histopathologic examination, finally diagnosed as angiosarcoma.

**Discussion:**

There is no published case of angiosarcoma onset of pericardial hematoma after repeated myocardial infarction. Multimodality imaging contributes to early diagnosis and optimal preoperative planning.

## Visual Summary

**Figure:**
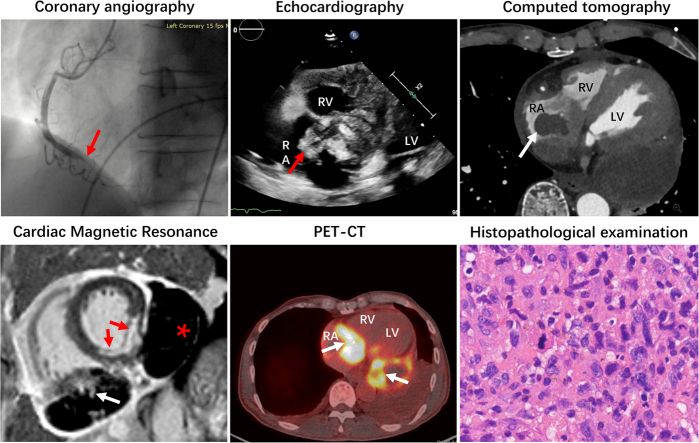

## History of Presentation

A 60-year-old male presented with unexplained repeated right coronary infarction, pericardial effusion, and hematoma, ultimately diagnosed as primary cardiac angiosarcoma (PCA). The patient had a history of 2 emergency percutaneous coronary interventions (PCIs) within the past year due to right coronary artery (RCA) myocardial infarction (Video 1), leading to the placement of 3 stents in the proximal, middle, and distal segments of the RCA.

**Take-Home Messages**

• This case highlights the significance of multi-modality imaging in assessing post–percutaneous coronary intervention pericardial masses.
• This case characterizes new clinical and imaging manifestations of cardiac angiosarcoma.

**Figure 1:**
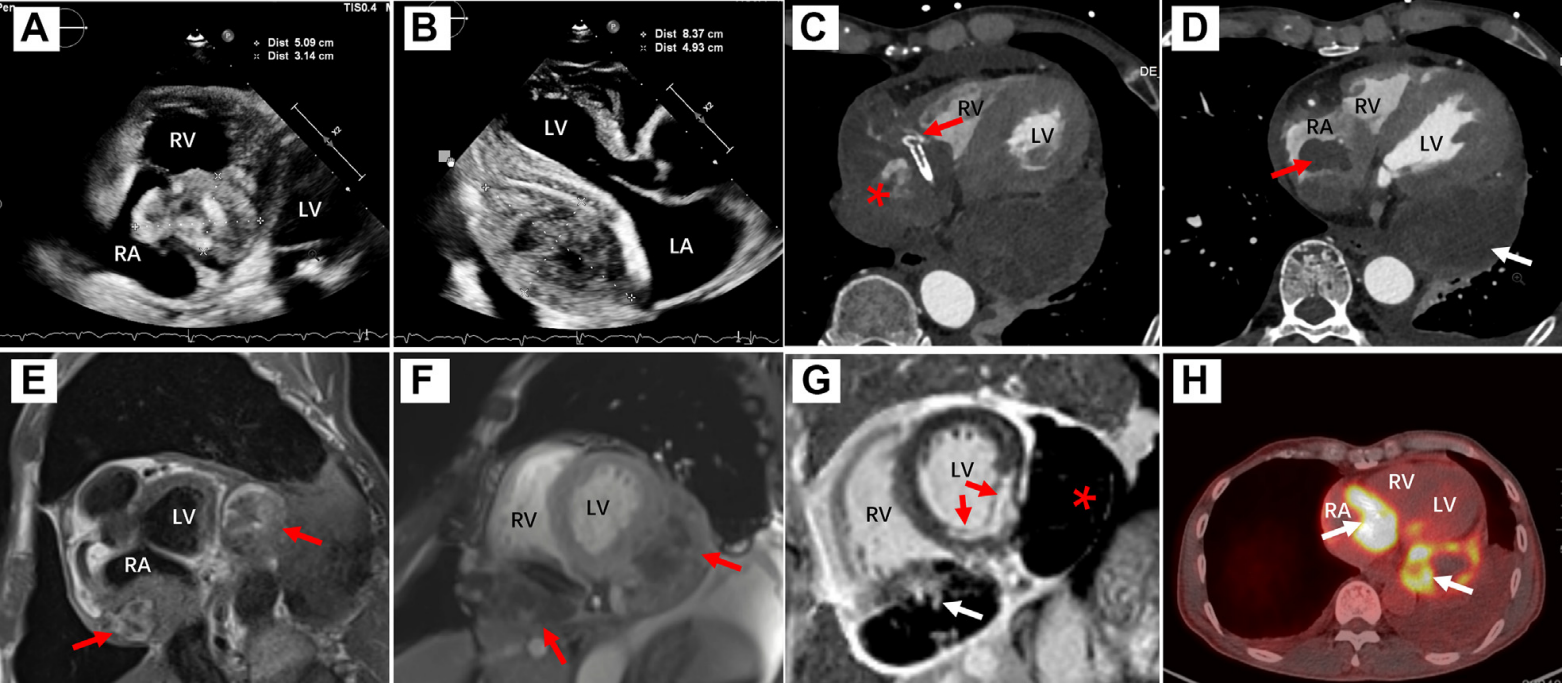
Multimodality Imaging of Cardiac Angiosarcoma TTE revealed a heterogeneous 5.09- × 3.14-cm mass located on the lateral wall of the RA (A) and an 8.37- × 4.93-cm hypoechoic mass on the lateral wall of the LV (B). CT showed the heterogeneous mass in the lateral wall of the RA encased the RCA, with stent angulation (C, red arrow) and contrast extravasation (C, asterisk), with partial invasion into the right atrial cavity (D, red arrow). The well-defined mass on the posterior wall of the LV had extensive pericardial adhesions, but without contrast enhancement (D, white arrow). T1-weighted imaging (E) and cine imaging (E) in CMR demonstrated that mixed T1 signal was noted in both masses (red arrows). LGEs were detected in the mass on the lateral wall of the RA (G, white arrow), but not in the mass on the posterior wall of the LV (G, asterisk). Transmural LGE was noted in the inferior septal and inferior wall (G, red arrows). PET-CT indicated increased FDG metabolism in both masses (H, white arrows). CT = computed tomography; CMR = cardiac magnetic resonance; FDG = fluorodeoxyglucose; LA = left atrium; LGE = late-gadolinium enhancement; LV = left ventricle; PET-CT = positron emission tomography–computed tomography; RA = right atrium; RCA = right coronary artery; RV = right ventricle; TTE = transthoracic echocardiography.

Two months after the second PCI, the patient experienced another episode of chest pain after emotional excitement with increased troponin T. Coronary angiography demonstrated significant stent angulation, in-stent filling defect, and contrast extravasation between the middle and distal RCA (Video 2). Transthoracic echocardiography (TTE) identified a severe pericardial effusion. Pericardial puncture was performed, draining 1,600 mL of bloody fluid. Cytologic examination of the pericardial fluid revealed no malignancy. One month later, the patient experienced fever and back pain. TTE revealed a heterogeneous 5.09- × 3.14-cm hematoma located on the lateral wall of the right atrium (RA), adjacent to the atrioventricular groove, with evidence of invasion into the RA cavity (Figure 1A). Additionally, an 8.37- × 4.93-cm hypoechoic hematoma with extensive pericardial adhesions was identified on the lateral wall of the left ventricle (LV), causing compression of the LV (Figure 1B, Video 3).

## Past Medical History

The patient had no history of cardiac disease.

## Differential Diagnosis

The differential diagnosis of a pericardial mass after PCI includes pericardial hematoma and thrombus. Imaging features indicative of malignancy include ill-defined borders, tissue planes invasion, or contrast enhancement on computed tomography (CT) or cardiac magnetic resonance (CMR), or increased fluorodeoxyglucose metabolism on positron emission tomography–computed tomography (PET-CT).

## Investigation

Cardiac contrast-enhanced CT demonstrated a heterogeneous mass on the lateral wall of the RA, encircling the RCA and partially invading the RA cavity. Stent angulation and contrast extravasation were observed within the mass. A well-capsule mass was identified on the posterior wall of the LV, with extensive pericardial adhesions but without contrast enhancement. CMR confirmed that lesion with an indistinct boundary involving the lateral wall of the RA displayed heterogeneous signal intensity on both T1-weighted and cine imaging sequences. The presence of contrast extravasation and late-gadolinium enhancement (LGE) suggested a composite mass comprising thrombotic material and neoplastic tissue. Additionally, the mass located on the posterior wall of the LV exhibited heterogeneous signal intensity on T1-weighted images. Cine imaging further revealed a core region with lower signal intensity compared with the peripheral rim. Notably, the absence of significant LGE within the entire mass supports the characterization of a central hematoma surrounded by organized thrombus. PET-CT further demonstrated increased fluorodeoxyglucose metabolism, which was noted in both masses, suggesting malignancy. Meanwhile, there was no evidence of distant metastasis (Figure 1).

## Management

**Figure 2:**
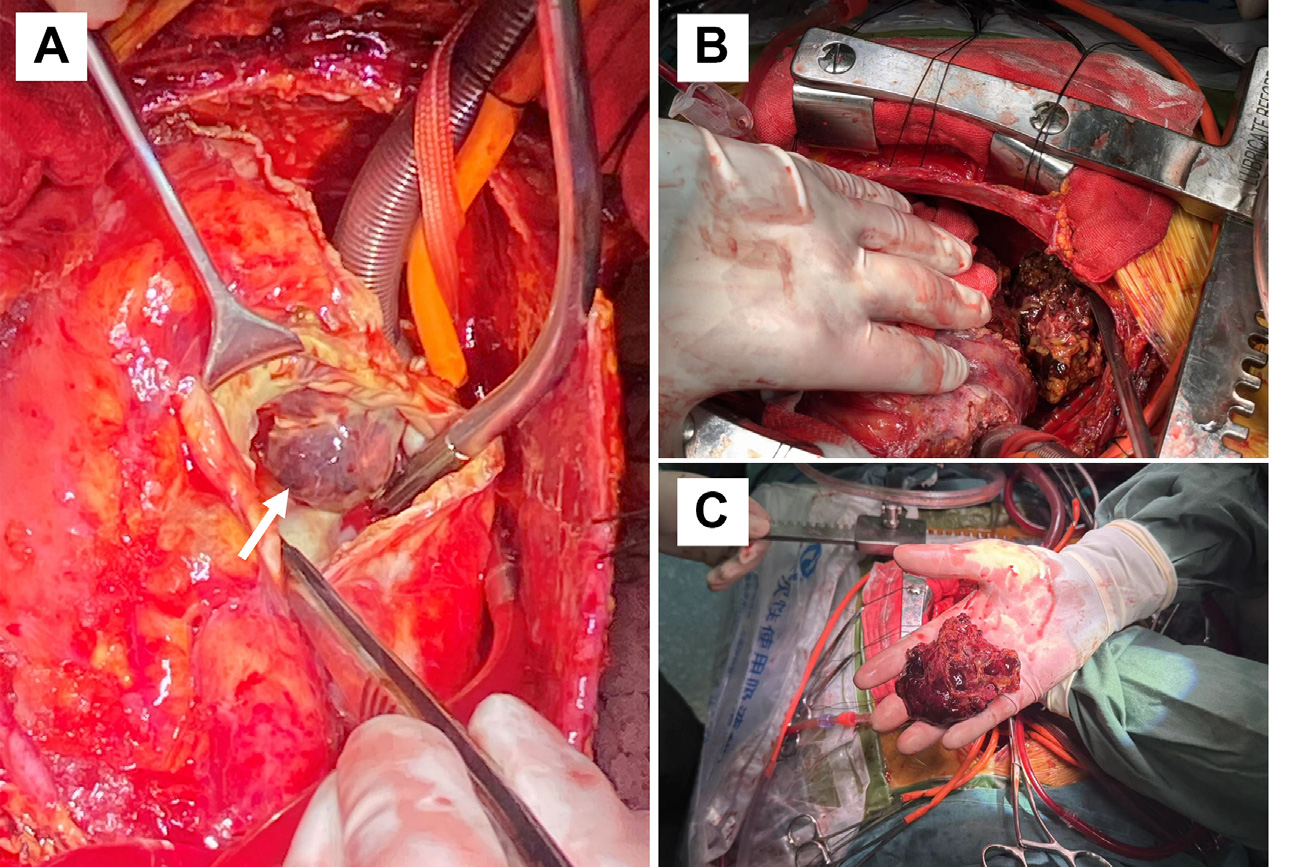
Intraoperative Imaging An intramural mass inside the RA connected with the subepicardial hematoma (A: white arrow) and a large volume of clotted blood on the posterior wall of the LV (B and C). Abbreviations as in Figure 1.

**Figure 3:**
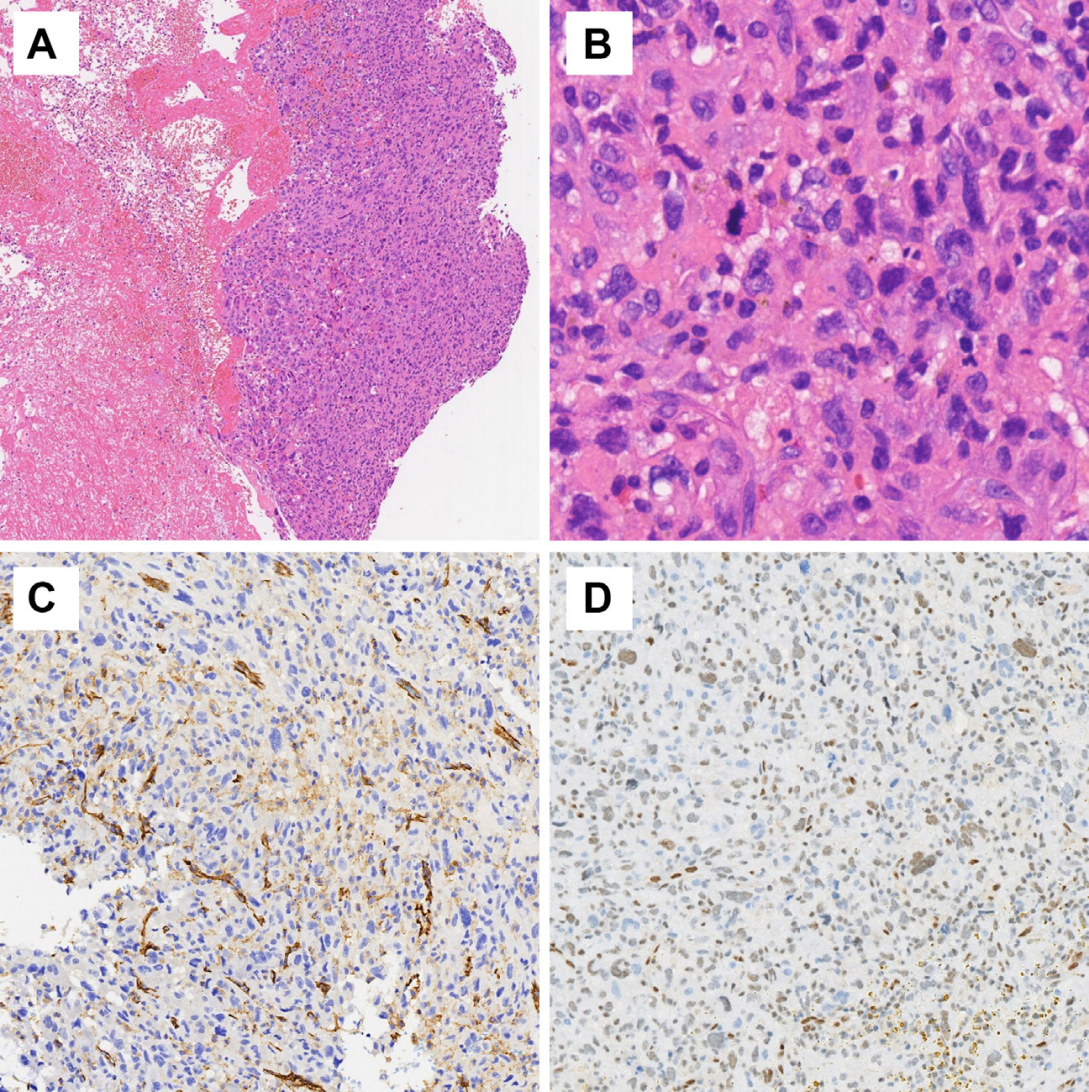
Histopathologic Examination H&E stains revealed small clusters of atypical cells, which were observed within extensive coagulative tissue (A, H&E ×10), with the presence of large, pleomorphic nuclei and frequent mitotic figures (B, H&E ×40). Immunohistochemical stains were positive for CD31 (C) and Fli1 (D). H&E = hematoxylin and eosin.

According to the abnormal multimodality imaging findings, a median sternotomy was performed to explore the etiology of the hemopericardium and to evacuate the hematomas. Intraoperative findings included extensive pericardial adhesions, exudation, myocardial edema, and a large amount of hemorrhagic fibrinous clots. On clearing the clots, 2 partial epicardial defects were identified. The first defect was located on the posterior wall of the RA along with a subepicardial hematoma penetrating the RA. This hematoma was connected to an intramural clot within the RA, which was excised after opening the RA (Figure 2A). The second defect was in the posterior wall of the LV, showing extensive clot formation without significant active bleeding (Figure 2B). Histopathologic examination of the excised tissue and clots revealed highly atypical cells with large, pleomorphic nuclei and frequent mitotic figures (Figures 3A and 3B). Immunohistochemical staining was positive for CD31 and Fli1 but negative for epithelial membrane antigen and smooth muscle actin, confirming a diagnosis of high-grade angiosarcoma (Figures 3C and 3D).

**Visual Summary:**
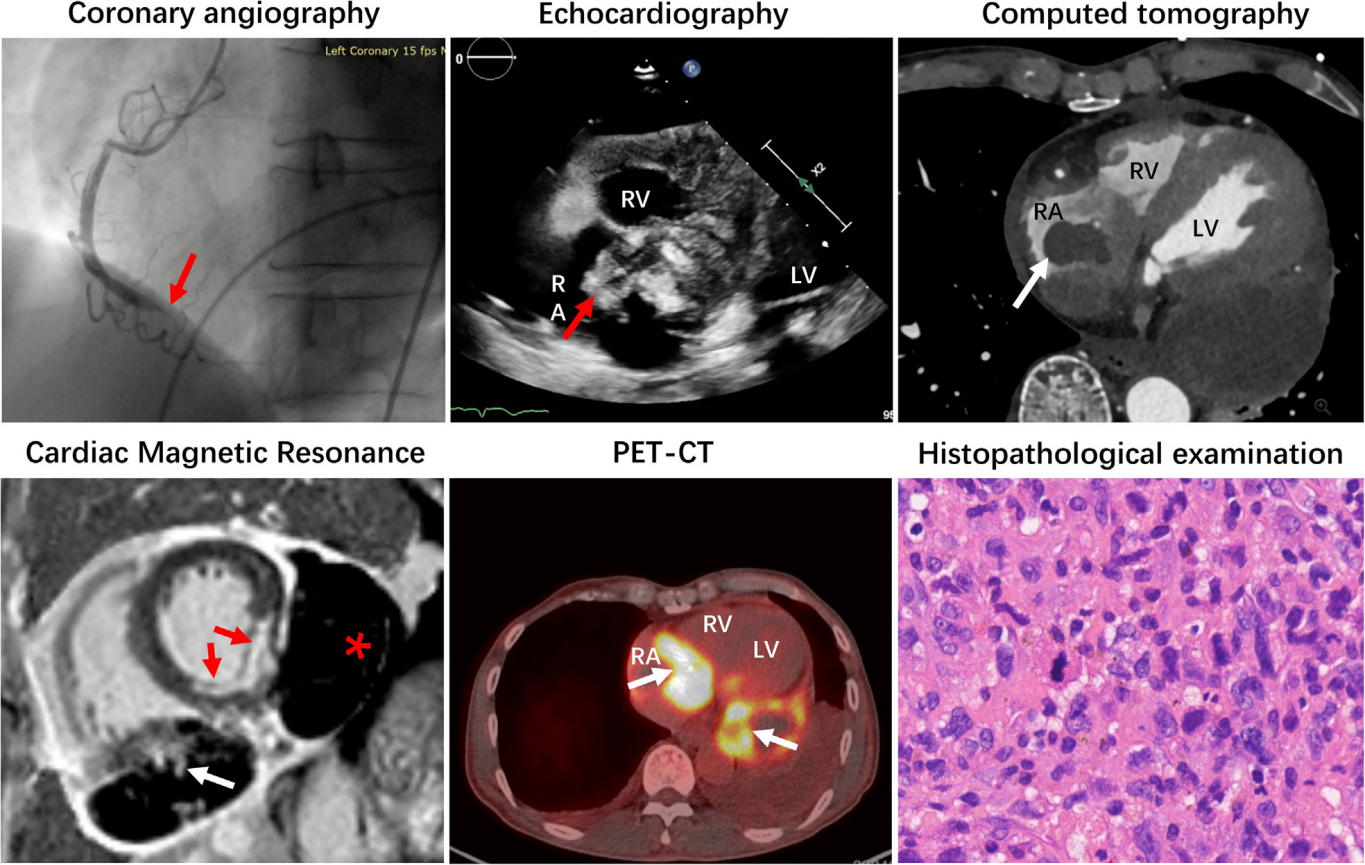
Multimodality Imaging in Diagnosing of Cardiac Angiosarcoma

## Outcome and Follow-Up

The patient underwent 2 cycles of chemotherapy consisting of albumin-bound paclitaxel (200 mg on day 1, 100 mg on day 8) and gemcitabine (1.4 g on days 1 and 8), followed by a 2-week rest period. Thirteen weeks after surgery, the patient died due to LV compression arising from the recurrence of the mass (12.8 × 7.0 × 9.8 cm) on the posterior wall of the LV.

## Discussion

We reported a case of PCA presented as pericardial hematoma after repeated right coronary infarction. The cause of repeated myocardial infarction and frequent hematoma formation remained unclear. Potential mechanisms included in situ cloth, thromboembolism originating from the tumor surface, or turbulence resulting from coronary compression due to the mass effect of the tumor. Multiple imaging modalities were used for early detection and assessment.

Primary cardiac malignant tumors are extremely rare. Angiosarcoma accounts for 25%-40% of sarcomas, making them the most prevalent type of cardiac sarcoma.^1^ They are 2-3 times more prevalent in men with a mean reported age of 41 years.^2^ Primary cardiac malignancies mainly affect the right side of the heart, particularly the RA, accounting for 75% of cases. Tumors originating from the left side of the heart were associated with a better prognosis.^3^ Angiosarcomas are often asymptomatic unless they reach a large size, at which point they are commonly associated with nonspecific cardiopulmonary symptoms due to tumor size and infiltration. Symptoms such as general malaise, fever, fatigue, and chest pain often precede the clinical signs and symptoms. A primary cardiac angiosarcoma typically manifests as chest pain, myalgia, dyspnea, palpitation, or clinical manifestation of pericardial effusion or tamponade, vena cava obstruction, arrhythmia, and chronic heart failure, which presents mostly as right-sided heart failure. About 80% of patients present with metastatic lesions at diagnosis, resulting in an average survival of <9 months, and the most common site of metastasis is the lung.

## Diagnostics and Imaging Modalities

Complementary imaging techniques are useful for diagnosis, evaluating disease extent, and detecting metastasis. TTE is sensitive in detecting intracardiac masses and can describe the tumor’s size, shape, attachment, mobility, its surrounding structures, and impact on flow dynamics, as well as complicating pericardial effusion. Transesophageal echocardiography offers high-resolution 2D ultrasound images and detailed blood flow illustrations, enabling clearer visualization of intracardiac structures and abnormal masses. On CT, angiosarcomas appear as irregular lobulated low-attenuation masses. CT is also capable of detecting potential metastasis. CMR offers more detailed contrast of soft tissues, characterization of infiltration. and extracardiac involvement. On T1-weighted imaging, the lesions appear isointense with multiple nodular areas of high-signal intensity, exhibiting a "cauliflower" appearance. LGE profile demonstrates heterogeneous enhancement encompassing a large unenhanced hemorrhagic and necrotic core.^4^ Pericardial invasion often presents as a "sunray" appearance, characterized by intense LGE along the tumor’s prominent vasculature.^5^ PET-CT provides critical information to determine the malignancy and to locate metastatic deposits, revealing irregular regions of high metabolic activity along the tumor. Coronary angiography should be performed to identify abnormal findings, which may include normal coronary arteries with a RA tissue blush, most commonly fed by a branch off the RCA.^6^ Identifying feeder vessels or coronary-to-atrium fistulae may help to avoid perioperative complications and to aid preoperative planning of atrial reconstruction.

Histopathology remains the gold standard for definitive diagnosis for PCAs, characterized by anastomotic vascular channels formed by malignant cells, solid spindle cells areas, and other regions primarily composed of anaplastic cells. Immunohistochemical stains for CD31, CD34, and factor VIII–related protein can be used to identify the endothelial origin of sarcomas.

## Treatment Approaches

Management of PCA is difficult due to its aggressiveness and poor prognosis. There is no standardized algorithm for managing PCA, with existing evidence derived primarily from case reports and single-center retrospective studies. Pathologic category, primary site, extent of invasion, and metastatic status should be considered in management strategies. However, evidence suggests surgical resection, including incomplete resection with positive surgical margins, accompanied by adjuvant chemotherapy, immunotherapy, or radiation therapy significantly improve the prognosis.^3^ Anthracycline-based regimens and taxane-based chemotherapy regimens with or without gemcitabine have been used in RCA.^7^ Although data is limited, immunotherapy, such as application of appropriate immune checkpoint inhibitors and dual-target drugs, has demonstrated notable efficacy in treating PCAs.^8^ Postoperative radiotherapy for cardiac malignancies remains challenging due to cardiac and respiratory movements that complicate efforts to minimize damage to adjacent tissues. Concurrent standard neoadjuvant chemotherapy may further exacerbate radiotherapy-induced cardiac damage. Recent advances in image-guidance techniques and stereotactic body radiation have enhanced dose sparing and successful application in treating cardiac tumors.^9^ Heart transplantation has been performed only on selected patients with PCAs. However, existing data suggest that the long-term survival of transplant recipients is comparable with those treated with other cancer therapies.^10^

## Conclusions

PCA is a rare and aggressive malignancy, posing significant challenges in both diagnosis and treatment. The unique advantages of echocardiography, CT, MRI, PET-CT, and angiography highlight the critical role of multimodal imaging for early diagnosis and optimal preoperative planning. Multimodality management, including any combination of surgery, chemotherapy, immunotherapy, and radiation therapy, has been associated with improved survival. Enhanced knowledge and understanding of PCA will lead to improved management and outcomes for this challenging condition.

## Funding Support and Author Disclosures

The authors have reported that they have no relationships relevant to the contents of this paper to disclose.

## Appendix

Video 1 Coronary Angiography Coronary angiography at the first acute myocardial infarction revealed a 75% stenosis with a long lesion in the proximal segment of the right coronary artery, and an acute total occlusion in the distal segment.

Video 2 The Last Coronary Angiography The last coronary angiography showed significant stent angulation, in-stent filling defect, and contrast extravasation between the middle and distal right coronary artery.

Video 3 TTE TTE demonstrated the hypoechoic mass on the lateral wall of the LV caused the compression of the LV.
